# Young male mating success is associated with sperm number but not with male sex pheromone titres

**DOI:** 10.1186/s12983-015-0124-y

**Published:** 2015-11-09

**Authors:** Tobias Kehl, Ian A.N. Dublon, Klaus Fischer

**Affiliations:** Zoological Institute & Museum, Greifswald University, Johann-Sebastian-Bach Str. 11/12, Greifswald, 17489 Germany; Evolutionary Ecology and Genetics Group, Biodiversity Research Centre, Earth and Life Institute, Université catholique de Louvain (UCL), Croix du Sud 4, Louvain-la-Neuve, 1348 Belgium

**Keywords:** Honest signals, Individual fitness, Intrasexual selection, Male produced sex pheromones, Pheromone titre quantification, GC-FID

## Abstract

**Background:**

Intraspecific communication is of crucial importance throughout the animal kingdom and may involve a combination of visual, gustatory, olfactory and acoustic cues. Variation in male sex pheromone amount and composition may convey important information to female conspecifics, for instance on species identity or age. However, whether increased male pheromone titres are associated with fitness benefits for the female, thus indicating a role as an honest signal, is under debate.

**Results:**

Against this background, we tested in the butterfly *Bicyclus anynana* (1) whether young males being successful or unsuccessful in gaining a mating differed in sex pheromone titres and (2) for associations between male pheromone titres and spermatophore mass, eupyrene sperm number, and a variety of female and offspring life-history traits. Successful and unsuccessful males did not differ in pheromone titres, however eupyrene sperm number was much higher in successful males. Pheromone titres were not associated with any fitness-related female or offspring trait measured in our study, though correlation analyses yielded evidence for trade-offs among specific traits. Patterns did not differ among control and olfaction-blocked females.

**Conclusion:**

Therefore, we suggest that in young *B. anynana* pheromone titres do not indicate male quality.

## Background

In animals the operational sex ratio is typically shifted towards males, i.e. within a given population there are usually more receptive males than females available at any point in time [1–3]. Consequently, male reproductive success is often limited by access to receptive females, while females are able to choose among several prospective partners [1–3]. Therefore, sexual selection in males often favours traits increasing the number of matings and / or sperm competitive ability [4, 5]. Correlates of male reproductive success are, for instance, traits related to the resource holding potential such as body mass, fat content, weaponry, persistence and aggressiveness [6–8] or sperm number and motility [9, 10].

In addition to male competitive ability, female choice is clearly important in determining male reproductive success [11, 12]. In fact, females may preferably mate with males showing a high resource holding potential / competitive ability which may directly benefit their offspring, or with males displaying specific cues typically being regarded as sexually selected signals [13, 14]. The latter may involve visual [15, 16], acoustic [17–19], olfactory [14, 20–22] or gustatory cues [22, 23]. If female mating decisions are at least partly based on sexual signals such as ornaments, the principal challenge from the female perspective is to reliably assess male quality based upon these cues. Which information may specific colour patterns or odours convey to the females, and are they associated with any direct or indirect fitness benefits? Several studies provide evidence for a covariation between sexually selected cues and male quality, thus suggesting honest signalling [15, 24, 25]. However, given that sexual conflict is widespread, such signals are prone to cheating as inferior males will nonetheless be selected to produce sexually attractive signals [26].

Sex pheromones are commonly used throughout the animal kingdom [27, 28] where they play an important role in conspecific recognition and mating decisions [29–33]. In addition to signaling species identity and age, sex pheromones may comprise honest signals in the sense that they provide information on mate quality. If so, the production of sex pheromones is predicted to be costly for which there is indeed some evidence [34–36]. In Lepidopteran insects, several studies revealed evidence that male sex pheromone (MSP) profiles may convey detailed information to potential mating partners [21, 37, 38], including male quality [39–41]. This has also been shown in Diptera [34].

Displaying a wide range of presumably sexually selected traits such as colour patterns or odour and undergoing rapid reproduction in laboratory environments, butterflies have become important models for sexual selection [16, 42, 43]. Importantly multiple traits like chemical and visual cues have been suggested to be involved in butterfly mating decisions [14]. Short-range communication may be facilitated by male produced pheromones [29, 44–46] though more work is needed to elucidate their role in male mating success [47]. Investigating such factors affecting male mating success is especially interesting in groups such as butterflies, lacking weaponry to inflict harm upon combatants but performing wars of attrition [8, 48, 49].

In the butterfly *Bicyclus* anynana it has been recently shown that males being successful in gaining a mating compared with unsuccessful males were characterized by higher MSP titres [20, 21, 43]. Male *B. anynana* have three MSPs (*Z*9-14:OH (MSP1), 16: Ald (MSP2) and 6,10,14-trime-15-2-ol (MSP3) [20]. Therefore, this species is a suitable model for exploring the basis of variation in sexual signals. Are MSP titres associated with male quality or any fitness benefits for the females? Consequently, are pheromones honest signals indicative of male qualities beyond species identity and age [21, 32]? Which benefits may females possibly derive from mating with males producing more sex pheromones, i.e. which traits might be associated with increased pheromone titres [34, 50]? Increased production of MSPs may also be associated with male condition, which may also increase competitive ability (such as more vigorous courtship) and thus mating success. The latter hypothesis rests on the assumption of a positive covariance between condition and sex pheromone production: males in a good shape may be able to both court more vigorously and produce more sex pheromones [51]. Of course, females may also prefer such males.

In principle, female preference for males with increased pheromone titres may be based on direct or indirect fitness benefits. Possible direct benefits in the *Bicyclus* system include the transfer of larger spermatophores, containing more accessory gland products and / or sperm, upon mating. Thus, a positive correlation between pheromone titre and spermatophore mass or sperm numbers is predicted. Additionally, females may receive indirect benefits by ensuring that their offspring inherits ‘good genes’ from the preferred males by e.g. providing a survival advantage [11, 52] or by the production of ‘sexy sons’ inheriting sexually attractive signals from the father, which will increase their own reproductive success (Fisherian run-away process; [26, 53]).

Based on this background we investigate associations between naturally occurring un-manipulated MSP titres in young males and potential direct and indirect fitness benefits for females of the butterfly *B. anynana*. We compare choice patterns among control and olfaction-blocked females. If females base their mate choice on pheromone titres, control females (with intact olfactory receptors in their antennae) should prefer males with high pheromone titres, while a lack of difference among groups would favour alternative hypotheses such as a prominent role of male vigour or traits other than pheromones. As proxies of direct fitness benefits we investigated spermatophore mass, the number of eupyrene sperm, and female longevity. Furthermore, we measured various offspring traits indicative of indirect fitness benefits.

## Results

### Experiment 1

No significant differences were found when comparing the number of successful males having higher or lower pheromone levels than their counterparts: MSP1 (control 26 versus 16: Χ^2^_1_ *=* 2.38; *p* = 0.1228; olfaction-blocked 23 versus 20: Χ^2^_1_ = 0.21; *p* = 0.6473), MSP2 (control 13 versus 21: Χ^2^_1_ = 1.88; *p* = 0.1701; blocked: 17 versus 22 Χ^2^_1_ = 0.64; *p* = 0.4233), MSP3 (control 24 versus 18: Χ^2^_1_ = 0.86; *p* = 0.3545; blocked: 20 versus 23: Χ^2^_1_ = 0.21; *p* = 0.6473), PC MSP (control: 17 versus 17: Χ^2^_1_ ≤ 0.01; *p* ≥ 0.9999; blocked 17 versus 22: Χ^2^_1_ = 0.64; *p* = 0.4233). Univariate comparisons revealed that in both treatments successful compared with unsuccessful males had significantly higher numbers of eupyrene sperm, whereas differences in spermatophore mass and MSPs were not significant (Table 1). Accordingly, generalised linear models revealed that eupyrene sperm number was the sole factor significantly differing between successful and unsuccessful males in both the female control (Wald-*Χ*^2^ = 10.24, *p* = 0.0014) and the olfaction-blocked group (Wald-*Χ*^2^ = 9.09, *p* = 0.0026). Correlation analyses revealed that spermatophore mass was significantly positively related to pheromone titres (except for MSP2) in both treatment groups (Table 2). Sperm numbers though were significantly positively related to pheromone titres (except for MSP2) in olfaction-blocked females only.

**Table 1:** Comparisons of spermatophore mass, eupyrene sperm number, MSPs 1–3, and the principal component extracted from MSPs 1–3 (means ± 1 SD) between successful and unsuccessful males having competed for a single virgin female, including ranges for MSPs 1–3 and the principal component, and results of paired t-tests

Trait	Successful		Unsuccessful		*DF*	*T*	*P*
a) Control	Mean ± SE	Range	Mean ± SE	Range			
Spermatophore mass [mg]	0.468 ± 0.091	0.14–0.63	0.439 ± 0.136	0.18–0.79	40	1.140	0.2610
Euyprene sperm [n]	7686.2 ± 3000.5	2560–15616	4583.0 ± 2828.1	768 - 11264	40	4.901	<0.0001
MSP 1 [ng * 350 μl^−1^]	1680.9 ± 673.2	463–2967	1487.1 ± 819.5	208–3534	41	1.444	0.1565
MSP 2 [ng * 350 μl^−1^]	87.2 ± 42.5	23–195	96.5 ± 49.8	32–216	33	−0.987	0.3306
MSP 3 [ng * 350 μl^−1^]	5556.6 ± 2780.2	1533–12212	5159.8 ± 2889.7	765–12710	41	0.784	0.4378
PC MSP	−0.0007 ± 0.975	−1.9–1.8	−0.0413 ± 1.074	−2.6–1-7	33	0.208	0.8365
b) Blocked							
Spermatophore mass [mg]	0.476 ± 0.090	0.25–0.65	0.457 ± 0.155	0.12–0.92	35	0.746	0.4609
Eupyrene sperm [n]	8732.4 ± 3429.6	2304–16384	4394.7 ± 2186.4	768–9728	35	6.990	<0.0001
MSP 1 [ng * 350 μl^−1^]	1726.7 ± 720.1	281–2832	1731.8 ± 903.7	259–3768	42	−0.038	0.9698
MSP 2 [ng * 350 μl^−1^]	83.3 ± 39.3	21–177	89.8 ± 41.6	23–163	38	−0.741	0.4632
MSP 3 [ng * 350 μl^−1^]	5595.3 ± 2959.9	1194–17842	5519.9 ± 3138.2	48–13555	42	0.149	0.8824
PC MSP	0.0067 ± 0.940	−3.4–1.4	−0.0823 ± 1.090	−2.8–1.7	38	0.526	0.6016

Data are presented separately for control and olfaction-blocked females. Significant *P*-values are given in bold

**Table 2:** Pearson correlations between MSPs 1–3 as well as the principal component (PC) extracted from MSPs 1–3 and spermatophore mass and eupyrene sperm number

a) Control	MSP 1	MSP 2	MSP 3	PC MSP
Spermatophore mass	0.4896	0.1984	0.5018	0.4692
	*P* < 0.001	*P* = 0.090	*P* < 0.001	*P* < 0.001
Sperm number	0.2019	0.1680	0.1172	0.1850
	*P* = 0.084	*P* = 0.152	*P* = 0.320	*P* = 0.115
b) Blocked				
Spermatophore mass	0.3106	0.1349	0.2946	0.3001
	*P* = 0.007	*P* = 0.252	*P* = 0.011	*P* = 0.009
Sperm number	0.2573	0.1284	0.2962	0.2761
	*P* = 0.027	*P* = 0.275	*P* = 0.010	*P* = 0.017

Given are correlation coefficients and *p*-values. Data are presented separately for control and olfaction-blocked females. Significant *P*-values are given in bold

### Experiment 2

The titres of the three MSPs were strongly correlated with each other and the resulting PC (Table 3), but not with any other trait measured except for marginal positive correlations of MSP2 and the pheromone PC with male pupal development time (Table 3). The latter correlations though are not significant after Bonferroni correction. However, several significant correlations were detected between female and offspring traits (Table 4). As the majority of these would not be significant after Bonferroni correction, we refrain from mentioning each significant correlation. Instead, we highlight patterns that may bear biological relevance. Longevity was positively related to lifetime fecundity. Early fecundity was positively related to lifetime fecundity, but negatively to pupal survival and several body size measures. Lifetime fecundity was positively related to male pupal time, but negatively to pupal survival rate and male wing length. Pupal survival rate was positively related to body size. Different size measures were generally positively correlated, as was the case for male and female pupal development time.

**Table 3:** Pearson correlations between MSPs 1–3 as well as the principal component (PC) extracted from MSPs 1–3 and an array of traits

No.	Trait	MSP 1	MSP 2	MSP 3	MSP PC
1	Female longevity	−0.0780	−0.0806	−0.0754	0.0815
		*P* = 0.492	*P* = 0.477	*P* = 0.506	*P* = 0.472
2	Early fecundity (until day 10)	0.0924	0.1211	0.0900	−0.1055
		*P* = 0.415	*P* = 0.285	*P* = 0.427	*P* = 0.353
3	Lifetime fecundity	0.1094	0.0951	0.0878	−0.1019
		*P* = 0.334	*P* = 0.401	*P* = 0.439	*P* = 0.369
4	Egg-hatching success	−0.0264	−0.0986	−0.0968	0.0769
		*P* = 0.816	*P* = 0.384	*P* = 0.393	*P* = 0.498
5	Larval survival rate	0.0891	0.1401	0.0820	−0.1079
		*P* = 0.432	*P* = 0.215	*P* = 0.469	*P* = 0.341
6	Pupal survival rate	−0.0531	−0.0375	−0.0384	0.0450
		*P* = 0.640	*P* = 0.741	*P* = 0.735	*P* = 0.692
7	Pupal mass (males)	−0.0216	0.0438	−0.0225	0.0007
		*P* = 0.849	*P* = 0.699	*P* = 0.843	*P* = 0.995
8	Adult mass (males)	−0.0377	−0.0660	−0.0163	0.0414
		*P* = 0.740	*P* = 0.561	*P* = 0.886	*P* = 0.715
9	Wing length (males)	−0.0379	0.0277	−0.0274	0.0137
		*P* = 0.739	*P* = 0.807	*P* = 0.809	*P* = 0.904
10	Pupal time (males)	0.2164	0.2506	0.1841	−0.2263
		*P* = 0.054	*P* = 0.025	*P* = 0.102	*P* = 0.044
11	Pupal mass (females)	−0.0299	0.0070	−0.0497	0.0258
		*P* = 0.792	*P* = 0.951	*P* = 0.661	*P* = 0.820
12	Adult mass (females)	0.0565	0.0472	0.0651	−0.0590
		*P* = 0.619	*P* = 0.678	*P* = 0.566	*P* = 0.603
13	Wing length (females)	−0.0306	0.0232	0.0183	−0.0035
		*P* = 0.788	*P* = 0.838	*P* = 0.872	*P* = 0.975
14	Pupal time (females)	0.1345	0.1929	0.1261	−0.1574
		*P* = 0.234	*P* = 0.087	*P* = 0.265	*P* = 0.163
15	MSP 1		0.8653	0.9281	0.9742
			*P* < 0.001	*P* < 0.001	*P* < 0.001
16	MSP 2			0.8227	0.9350
				*P* < 0.001	*P* < 0.001
17	MSP 3				0.9599
					*P* <0.001

Traits 1–4 were measured in female mating partners, traits 5–14 in the respective females’ offspring, and traits 15–17 in male mating partners. Given are correlation coefficients and *p*-values. Significant *P*-values are given in bold

**Table 4:** Pearson correlation matrix among various traits measured in females (traits 1–4) and their offspring (5–14)

	2	3	4	5	6	7	8	9	10	11	12	13	14
1 Female longevity	0.2017	0.0018	0.4543	0.0741	−0.2062	0.1700	−0.0215	0.1730	0.1261	0.1524	0.0785	0.0456	0.0395
	*P* = 0.073	*P* = 0.987	*P* < 0.001	*P* = 0.513	*P* = 0.066	*P* = 0.132	*P* = 0.850	*P* = 0.125	*P* = 0.265	*P* = 0.177	*P* = 0.489	*P* = 0.688	*P* = 0.728
2 Early fecundity (until day 10)		0.0816	0.8082	−0.1074	−0.3382	−0.2714	−0.1278	−0.4017	0.1784	−0.4408	−0.2509	−0.3486	0.0867
		*P* = 0.472	*P* < 0.001	*P* = 0.343	*P* = 0.002	*P* = 0.015	*P* = 0.259	*P* < 0.001	*P* = 0.113	*P* < 0.001	*P* = 0.025	*P* = 0.002	*P* = 0.444
3 Egg-hatching success			0.0629	−0.0265	−0.0220	−0.0837	−0.0647	−0.1766	0.0533	0.0695	0.1178	−0.0200	0.0643
			*P* = 0.579	*P* = 0.815	*P* = 0.846	*P* = 0.460	*P* = 0.568	*P* = 0.117	*P* = 0.639	*P* = 0.540	*P* = 0.298	*P* = 0.860	*P* = 0.571
4 Lifetime fecundity				−0.0526	−0.3188	−0.1262	−0.0895	−0.3135	0.3264	−0.2072	−0.1096	−0.2189	0.1460
				*P* = 0.643	*P* = 0.004	*P* = 0.265	*P* = 0.430	*P* = 0.005	*P* = 0.003	*P* = 0.065	*P* = 0.333	*P* = 0.051	*P* = 0.196
5 Larval survival rate					−0.1533	−0.1960	−0.2666	0.0069	0.0837	−0.0637	−0.1112	0.0498	0.1242
					*P* = 0.175	*P* = 0.081	*P* = 0.017	*P* = 0.951	*P* = 0.460	*P* = 0.575	*P* = 0.326	*P* = 0.661	*P* = 0.272
6 Pupal survival rate						0.2968	0.3201	0.2692	−0.0662	0.3484	0.1911	0.2899	−0.1917
						*P* = 0.008	*P* = 0.004	*P* = 0.016	*P* = 0.559	*P* = 0.002	*P* = 0.090	*P* = 0.009	*P* = 0.088
7 Pupal mass (males)							0.5860	0.7311	0.0890	0.5120	0.2440	0.3823	−0.0952
							*P* < 0.001	*P* < 0.001	*P* = 0.432	*P* < 0.001	*P* = 0.029	*P* < 0.001	*P* = 0.401
8 Adult mass (males)								0.3006	−0.1226	0.2264	0.1365	0.1468	−0.1861
								*P* = 0.007	*P* = 0.279	*P* = 0.043	*P* = 0.227	*P* = 0.194	*P* = 0.098
9 Wing length (males)									−0.0756	0.4754	0.2755	0.4204	−0.0584
									*P* = 0.505	*P* < 0.001	*P* = 0.013	*P* < 0.001	*P* = 0.607
10 Pupal time (males)										−0.0236	−0.0705	−0.1476	0.4015
										*P* = 0.835	*P* = 0.534	*P* = 0.191	*P* < 0.000
11 Pupal mass (females)											0.6358	0.6490	0.1671
											*P* < 0.001	*P* < 0.001	*P* = 0.139
12 Adult mass (females)												0.5876	0.0345
												*P* < 0.001	*P* = 0.761
13 Wing length (females)													−0.0452
													*P* = 0.690
14 Pupal time (females)													

Given are correlation coefficients and *P*-values (without Bonferroni correction). Significant *P*-values are given in bold

## Discussion

In our model system the operational sex ratio is clearly shifted towards males, as female *B. anynana* typically mate only 1–2 times within their lifespan while males are able to mate multiple times [54, 55]. Therefore, sexual selection is predicted to favour increased male competitive ability while females are predicted to be the choosy sex. Indeed, evidence suggests that both male aggressiveness and willingness to persist and female choice contribute to male mating success in *B. anynana* [13, 56–58]. For instance, female *B. anynana* were found to often reject courting males [20, 57].

**Fig. 1:**
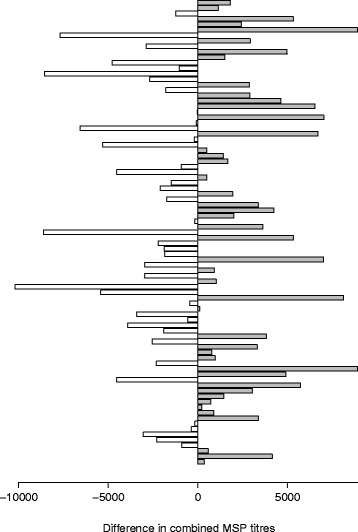
Variation of combined male sex pheromone titres (sum of MSPs 1–3) for pairs of *Bicyclus anynana* males having competed for a single female (*n* = 85). Each bar represents the MSP titre difference for one pair of males, with negative values (open bars) indicating that unsuccessful males had a higher combined MSP content than their successful counterparts and positive values (filled bars) the opposite. Data are presented for both control and olfaction-blocked females

In this study, experiment 1 did not reveal evidence for a decisive role of naturally occurring variation in pheromone blends of young males for female mating decisions, as (1) males with higher pheromone titres were not more successful and (2) successful and unsuccessful males did not differ in pheromone titres. While a lack of differences was expected for the olfaction-blocked females, the control females did in principle have the possibility to discriminate between males based on pheromone titres, which was not the case. Females not discriminating on the basis of pheromone titre contrasts with the findings of [14, 21, 43]. An important difference between our recent and the above studies having found positive effects of pheromone titres in *B. anynana* is that here we exclusively and deliberately investigated naturally occurring MSP variation in young males only (Fig. 1). In [43], for instance, pheromone titres were experimentally manipulated by surgically removing the androconia and afterwards perfuming males with different synthetic pheromones blends. Differences in pheromone titres among random wild-type males are likely to be much smaller than those induced by manipulative conditions, and may thus be more realistic from an ecological perspective. Hence, positive results based on experimental manipulations [14, 21, 43, 57, 58] may indicate an important role of sex pheromones in species recognition rather than intraspecific female choice. This notion is supported by the fact that olfaction-blocked females are generally much more reluctant to mate when compared with control females, and that control females strongly discriminate against largely pheromone-deprived males [14, 57]. In this work, we tested young males which were certainly sexually mature, but had relatively low MSP titres specifically with regard to MSP2 (hexadecanal) [43]. Therefore, future studies should test whether the patterns found here also hold in older males, exhibiting higher MSP titres. Another potential source of variation not covered by our study is individual variation in pheromone release rates during courtship. Indeed quantifying volatile MSP release from the courting male [44] would add useful information here.

Interestingly successful males transferred substantially higher eupyrene sperm numbers to females compared with unsuccessful males, suggesting that mating success is not random despite a lack of observed differences in pheromone blend. Note that unsuccessful males were mated in groups with many males and females. Thus, the perceived risk of sperm competition should be high in unsuccessful males such that a lower risk of sperm competition cannot explain their reduced sperm numbers. In the dipteran *Drosophila pseudoobscura*, for instance, the presence of mating rivals alters the copulation duration and increases the number of sperm being transferred upon mating [59]. Similar results were obtained in the butterfly *Pieris napi* in which males increased the size of their transferred ejaculate under increased male density, with male sex pheromones being the cue used by males to asses male density and the associated risk of sperm competition [60]. Though the magnitude of ejaculate increase under high male density differed among generations, the general pattern remained [61]. Alternatively, unsuccessful males may have reduced their reproductive investment to save sperm for future mating opportunities when being faced with a higher number of females [62].

However, as sperm numbers / ejaculates were hitherto mostly found to be increased rather than decreased under high densities and because mating couples were removed from the mating cages immediately after the initiation of mating in our study, social influences are unlikely to be the reason for reduced sperm numbers in unsuccessful males. Our results suggest that when young male *B. anynana* present themselves for mating the role of male sex pheromones is smaller than initially thought. The most straight-forward explanation for our results seems to be that successful males generally have a better body condition. This interpretation is in line with an earlier study having found that successful as compared with unsuccessful males show a higher fat content, longer wings, a heavier thorax and higher phenoloxidase expression levels [63]. A better condition may in turn allow for both more vigorous courtship and the production of more sperm. Whether the higher mating success of males in a better shape is a direct consequence of a more vigorous courtship or female preference for such males is currently not clear.

Interestingly sperm numbers differed among successful and unsuccessful males while spermatophore mass did not, supporting the notion that the latter may be a poor proxy of male quality [64]. Male *B. anynana* may cheat females by increasing the water content of the spermatophore, which may delay female remating and thus reduce the risk of sperm competition [65].

Although only eupyrene sperm numbers differed among successful and unsuccessful males, spermatophore mass and euyprene sperm numbers (the latter in olfaction-blocked females only) were positively related to MSP titres. The fact that such correlations did not result in a significant variation in pheromone blends suggests that the latter is of subordinate importance only in our experiment. This notion is further supported by the data derived from our no-choice experiment. No significant correlation between male pheromones and any trait investigated was found, suggesting that pheromone titres do not provide reliable information on male quality in young *B. anynana* males. Thus, if females would prefer males with higher titres, they would not be able to derive fitness benefits, at least not with regard to the traits investigated here.

Taken together our findings suggest that in young males, condition and perhaps courtship vigour are important for mating success, while we found no evidence for a significant role of natural variation in MSPs within one age class for female mating decisions. This is in line with earlier findings suggesting a prominent role of male behaviour in this species [43, 56, 57, 66]. For instance, old male mating advantage persisted in spite of a manipulation of female olfactory reception or male pheromone blend, presumably based on their more aggressive behaviour [43, 57]. This notion is further supported by the fact that females do not reject younger males more often than older males [57]. Moreover, we found no evidence that male produced pheromones are associated with any fitness-related trait measured in either females or their offspring. Of course, our findings do not rule out that male produced sex pheromones may be associated with other fitness-related parameters not investigated here. As mentioned previously, quantifying volatile MSP release from the courting male into the surrounding environment would shed further light on our results.

Experiment 2 revealed several correlations across life-history traits. Although testing for such correlations was not the principal aim of our experiment, we would like to highlight at least some patterns presumably bearing biological significance. As expected, lifetime fecundity was strongly related to early fecundity, indicating that the majority of eggs are laid early within the oviposition period [55]. Lifetime fecundity was positively related to longevity, indicating that females living longer produce more eggs [64, 67]. Both early and lifetime fecundity tended to negatively affect pupal development time and survival as well as offspring body size. These findings clearly suggest trade-offs between offspring quality and quantity [68]. The positive correlations between pupal (and larval) survival and measures of body size support the widely held notion of fitness benefits being conferred to large individuals [69, 70].

## Conclusions

We conclude that in young male *B. anynana* male sex pheromones do not seem to function as an honest signal, in the sense of indicating intraspecific variation in male quality beyond species identification and age. We suggest that at least for young males first encountering a female the role of male sexual pheromones in conveying information on male quality is limited, which may not necessarily be the case in older experienced males that have survived predation. Most results testing for an effect of MSPs on mating success cannot rule out that patterns arise from (1) a vital role of MSPs in species recognition [21, 32, 37, 56, 57] or (2) a positive correlation of MSPs with overall condition, such that males in a good shape may be able to both court more vigorously and at the same time to produce more sex pheromones [51]. Thus, variation in condition rather than pheromone titres may truly affect mating success. Future experiments should aim at disentangling these alternative hypotheses.

## Methods

### Study organism and rearing conditions

The Squinting Bush Brown *B. anynana* (Butler, 1879) is a nymphalid, fruit-feeding butterfly, whose distribution ranges from southern Africa to Ethiopia [71]. It exhibits striking phenotypic plasticity with two seasonal morphs, as an adaptation to alternate wet-dry seasonal environments and the associated changes in resting background and predation [72]. Reproduction takes place during the warmer wet season when oviposition plants are abundantly available, and where 2–3 generations occur. Reproduction ceases during the colder dry season in which butterflies do not mate before the first rains at the beginning of the next wet season [73]. A laboratory stock population was established at Greifswald University, Germany, in 2008 from several hundred eggs derived from a well-established stock population at Leiden University, The Netherlands. The Leiden population was founded in 1988 from 80 gravid females caught at a single locality in Malawi. In each generation several hundred individuals are reared maintaining high levels of heterozygosity at neutral loci [74]. For this study butterflies from the Greifswald stock population were used.

### Experimental design

We performed two experiments to test for associations between MSP titres and other male traits and collected approximately 1000 eggs for each. Larvae were reared in population cages on potted maize plants under constant conditions at a 12/12 h light–dark cycle, 27 °C and 70 % relative humidity. The conditions used are similar to those at which *B. anynana* develops and reproduces during the favourable wet season in the field [54, 73]. Resulting pupae were collected daily and transferred to cylindrical hanging cages. Following eclosion, individuals were separated by sex and eclosion day in order to avoid mating prior to experiments. Throughout the experiments all butterflies were supplied with moist banana and water enabling feeding *ad libitum*.

In experiment 1 we examined differences in MSP titre, spermatophore mass, and eupyrene (fertile) sperm number between successful and unsuccessful males. We performed 110 mating trials, 91 of which were successful in gaining a mating within 6 h. In each trial two 2-day old males competed for either a control or an olfaction-blocked 2-day old female in a cylindrical hanging cage (30 cm diameter, 15 cm height). As mentioned above, male *B. anynana* butterflies have three MSPs which have been shown to vary with male age [21]. While hexadecanal (MSP2) is expressed at low on-wing concentrations in young individuals such as the ones tested here, it was previously shown that females having mated with a younger as compared with an older male, have a fitness advantage [56]. Therefore, although the latter generally show higher levels of pheromones [43], we decided to investigate natural variation in pheromone titres in young rather than old males. Although the usage of relatively small cages is suspected to exacerbate male-male competition [75], they allow for high levels of comparability, reproducibility, and were not found to affect female polyandry or old male mating advantage in earlier experiments [66, 76]. Additionally, even in a large tropical insectary space, there are often space constraints.

One day prior to the respective mating trial, olfaction-blocked females were treated with a transparent, quickly drying nail polish (Essence; Colour & Go, Cosnova GmbH, Sulzbach, Germany) on the club surface of their antennae [14]. To control for confounding solution effects, the control group received a sham-treatment by applying nail polish on the right anterior forewing as opposed to the antennae. Cages were monitored for a maximum of 6 h or until a mating occurred. All the time prior to mating trials, all males were housed group-wise in spacious cages (maximum 30 individuals per cage). Unsuccessful males (*N* = 91) were afterwards (i.e. on the same day) mated in groups with at least as many randomly chosen 2–3 days old females to obtain their spermatophores. In order to avoid possible negative effects of the presence of male rivals on the number of sperm being transferred [59], couples were removed from the cage immediately after mating had commenced. In order to limit further MSP volatilisation and to prevent sperm cells from dispersing out of the bursa copulatrix into the spermatheca, all males and females were placed into glassine envelopes and placed into a container (Air Liquide, Voyageur 12), cooled with liquid nitrogen, immediately after mating and subsequently stored in a freezer at −80 °C.

In experiment 2 we investigated associations between MSPs and female and offspring traits to test for direct and indirect fitness benefits associated with increased pheromone titres. We successfully performed 100 mating trials (out of 122 trials), in each of which one random 2-day old virgin male and one random 2-day old virgin female were set up for mating per cage (no-choice assays). As above, males were frozen immediately after mating had ceased, and their wings were subsequently used to measure MSPs. Females were, in contrast to above, set up individually for egg-laying in 1 L translucent plastic pots containing a maize leaf as an egg-laying substrate and moist banana for feeding [77–79]. The first ca. 30 eggs produced per female were used to score egg-hatching success. The other eggs were transferred, separated by female, to elongated sleeve-like gauze cages. Each ‘sleeve’ cage thus contained one full-sib family (*N* = 87). Thirteen females produced no offspring or were lost during egg-laying. Larval density was standardised to a maximum of 30 larvae per sleeve. We scored female longevity, lifetime fecundity, and egg hatching success as well as the following offspring traits: larval and pupal survival, pupal development time, pupal mass, adult mass, and wing size. To investigate egg-hatching success, eggs were transferred to petri dishes containing moist filter paper in order to prevent desiccation. Eggs were checked daily until no more larvae hatched for at least 48 h. Pupal mass was measured 1 day after pupation to the nearest 0.01 mg using a microbalance (Kern ABJ 120-4 M). To score adult mass, butterflies were frozen 1 day after eclosion and afterwards weighed as well. Wings were photographed with a digital camera (Leica DC300) connected to a stereo microscope (Leica M275) to subsequently measure forewing length using NIS Elements software (Nikon Instruments).

### Quantification of MSPs

Male wings were used for pheromone extractions following established protocols for this species [20, 22, 80]. For each male, one fore and one hind wing were carefully removed from the thorax using dissection scissors. Afterwards, wings were submerged for 10 min in 350 μL hexane (98 %, HPLC grade) containing an internal C15 standard (10 ng μl^−1^ trans-4-tridecenyl acetate (Sigma Aldrich)).

For pheromone chromatography and quantification we used a gas chromatograph (Agilent GC7890A) in conjunction with a flame ionisation detector (Agilent Technologies, Belgium; GC-FID). A 30 μm x 320 μm x 0.25 μm DB-5 phase column (Agilent, 19091 J-413) was run in constant flow mode with laboratory generated H_2_ carrier gas. In the 20-minute temperature program, the initial temperature of 75 °C was held isothermally for 3 min, then ramped at 20 °C min^−1^ until 220 °C, after which the ramping rate was increased to 30 °C min^−1^ until 300 °C. The final temperature was held constant for 7 min. The FID was heated to 250 °C with H_2_ flow set to 30 ml min^−1^, air (Standard air, Praxair, Schoten, Belgium) at 350 ml min^−1^, and N_2_ (Praxair) makeup at 20 ml^−1^. Hydrogen was generated from high purity distilled water (Barnstead Easy Pure II, Thermo Fisher Scientific, Erembodegem Belgium) using a Peak PH300 gas generator (Peak Scientific, Inchinnan, Scotland).

Hexane samples containing extracted pheromones were injected into the GC-FID using a 7693 ALS autosampler (Agilent), injecting 1 μl. Injections were made in splitless mode and samples were deposited into a 2 mm quartz direct injection liner (Agilent 518–8818) providing 250 μl volume. Injector temperature was held at 250 °C and 14.23 psi with a septum purge flow of 3 ml min^−1^, and a purge time of 1.5 min at 40 ml min^−1^. As previously stated, male *B. anynana* butterflies have three male sex pheromones (*Z*9-14:OH (MSP1), 16: Ald (MSP2) and 6,10,14-trime-15-2-ol (MSP3) [20]. Under the chosen conditions trans-4-tridecenyl acetate eluted on average at 7.74 min, MSP1 at 7.57 min, MSP2 at 8.39 min, and MSP3 at 8.56 min. MSP retention times were confirmed through injection of a 1:1:1 pheromone mixture [3 ng μl^−1^] prepared from external standards (kindly synthesized at Mittuniversitetet, Sundsvall, Sweden). All acquisitions and integrations were conducted with GC Chemstation B.04.03-SP2 (105) (Agilent). No column compensation algorithms were used as bleed was insignificant during the relevant portion of the temperature cycle.

### Analyses of spermatophore mass and eupyrene sperm cells

To analyse spermatophore mass and eupyrene sperm numbers, females were thawed and dissected in Ringer’s solution [81]. The bursa copulatrix, which contains the male spermatophore, was removed. Surplus Ringer’s solution was removed from the bursa using filter paper, and afterwards the bursa was weighed on an electrobalance (Sartorius LE225D) to the nearest 0.01 mg. Thus, the mass of the bursa copulatrix containing the spermatophore was used as a proxy of spermatophore mass, as the mass of the bursa is negligible [82]. After weighing, the spermatophore was transferred to a cavity slide with a droplet of Ringer’s solution, opened with forceps and stirred gently to disperse the sperm. Eupyrene (fertile) sperm bundles were counted within the cavity slide using a microscope (Zeiss ICS KF2) at 40x. Additionally butterflies have infertile apyrene sperm, which are much smaller than eupyrene sperm and cannot be seen at the chosen magnification [82]. To achieve the absolute number of eupyrene sperm the number of bundles was multiplied by 256, owing to the fact that in Lepidoptera all the sperm in a bundle originate from a single spermatogonium, which undergoes a fixed number of 8 divisions [82–84].

### Statistics

In experiment 1, we first scored the number of successful males having higher MSP levels than their counterparts and vice versa. We tested the resulting numbers against even distributions using chi-square tests. Subsequently, differences between successful and unsuccessful males in the traits measured were analysed using paired t-tests. Afterwards, generalized linear models with binomial error distribution and logit-link function were constructed for both control and olfaction-blocked female groups. As MSP1, MSP2, and MSP3 were highly correlated to each other (all pairwise *r*-values > 0.45; *p* < 0.001) we performed a principal component analysis for each data set to reduce the number of interrelated variables. For further analyses we used in each case the first principal component (PC). PC1 had an eigenvalue of 2.19 (all other eigenvalues < 0.58) and explained 73.1 % of the total variation in the control group, and an eigenvalue of 2.16 (all other eigenvalues < 0.64) and explained 72.1 % of the total variation in the olfaction-blocked female group. Furthermore, to account for the statistical dependency of the data derived from individual mating trials, we calculated the difference between successful and unsuccessful males for each trait, thereby generating a single value per male pair. One male of each trial was randomly defined as the ‘focal’ individual, i.e. the one from which the values of the opponent were subtracted. This procedure yielded difference values for each mating trial and trait, which were subsequently used in the generalised linear models by encoding unsuccessful focal males with ‘0’ and successful focal males with ‘1’. Models were constructed based on spermatophore mass, sperm number, and the PC reflecting spermatophore titres by stepwise forward inclusion of significant factors. Pearson correlations were used to investigate correlations between sex pheromones and other traits.

To analyse the data obtained in experiment 2 we used Pearson correlations testing for associations between MSPs and female and offspring traits. As above we performed a principal component analysis based on the three male sex pheromones. We used the resulting first PC for correlation analyses, having an eigenvalue of 2.7 (all other eigenvalues < 0.7) and explaining 90.9 % of the total variation in MSPs. We additionally computed a Pearson correlation matrix involving all female and offspring traits. All statistical analyses were performed using Statistica 8.0 (StatSoft Inc.).

### Availability of data and materials

The data set supporting the results of this article is available in the Dryad repository: doi:10.5061/dryad.670nh.
